# Editorial: Regenerative medicine in neurodegenerative diseases and aging: challenging the redox homeostasis

**DOI:** 10.3389/fnins.2023.1238781

**Published:** 2023-06-27

**Authors:** Elisabetta Mormone, Eugenio Luigi Iorio

**Affiliations:** ^1^Unitá Produttiva per Terapie Avanzate, IRCCS Casa Sollievo della Sofferenza Hospital, San Giovanni Rotondo, Apulia, Italy; ^2^International Observatory of Oxidative Stress, Salerno, Italy

**Keywords:** hyaluronic acid, neurogenesis, Nrf2, regenerative medicine, ROS, senescence, sirtuins

The aim of this Research Topic is to bring together the scientific evidence related to the multiple roles played by reactive oxygen species (ROS) in regenerative medicine for neurological diseases. It is now clear that the redox system is a ubiquitous homeostatic system whose primary purpose is to allow an organism, cells, or tissues to react to environmental challenges (intra- and extra-cellular) through the activation of adaptive mechanisms, aimed at survival, based on redox reactions. Recently, the positive role played by ROS in tissue healing and repair has also been emerging. Thus, under this light, dampening ROS levels can potentially inhibit normal healing, but on the other hand, pathologically high levels of ROS can cause a decline in the regenerative process. In fact, in this delicate balance, the redox system provides for the reversible or irreversible oxidation of molecular targets as transcription factors; among these, nuclear factor erythroid 2-related factor 2 (Nrf2) has widely been described as one of the main transcription factors involved in the maintenance and adaptation of intracellular redox homeostasis together. In this context, non-radical species such as hydrogen peroxide (H_2_O_2_) or singlet molecular oxygen, rather than free-radical species, perform major second messenger functions. As showed by several studies, Nrf2 exerts its primary function under physiological ranges of stress exposure through conditions of oxidative eustress to restore the “golden mean” and is thus essential for functional redox signaling. Moreover, Nrf2 is acetylated by sirtuins that would act as metabolic sensors, as described by Mormone et al. in this Research Topic. Therefore, pro-oxidative shifts are essential for the physiology and maintenance of cells or tissues and the occurrence and/or production of endogenous oxidants, which also but not exclusively include ROS; this is not harmful *per se*, but it fulfills important physiological functions. In order to maintain this balance, Nrf2 regulates the transcription of a variety of genes encoding for proteins involved in many different cellular processes such as metabolism, cell cycle regulation, and regeneration. In their review included in this Research Topic, Angelopoulos et al. highlighted that Nrf2 plays a pivotal role in the regulation of neural stem cells (NSCs) fate, acting on mitochondrial-to-nuclear retrograde signaling, which enables extensive communication between the mitochondria and the nucleus, influencing metabolism, stemness, and survival. In this review and in the other mentioned above by Mormone et al., the authors strengthen the new concept that the metabolic shift, from glycolysis to oxidative phosphorylation, is a mechanism to drive NSCs fate decisions. The data reported in the scientific work included in this Research Topic also show the role that SIRT3/AMPK/Nrf2 pathway has in relieving the oxidative stress response after treatment with transcranial near-infrared laser in aged mice with postoperative neurocognitive disorder (Zhong et al.).

Oxidizing species such as H_2_O_2_ or hydroxyl radical can also target pathogens in order to neutralize potentially toxic agents, as observed in the macrophages and mast cells defense activity. When oxidizing species activate transcription factors, and when they act to neutralize toxic agents, it is fundamental a return to the basal condition through the intervention of reducing species, capable to return, when possible, the electron to the molecular target and, possibly, to the oxidizing species (if in excess). When the adaptive response of the redox system fails, it triggers a condition of “oxidative di-stress” that can be associated with a condition of chronic silent inflammation or proceed independently of it, while inflammation is always associated to oxidative distress. The clinical consequence of this condition is premature aging, driven by the cellular senescence mechanism, and numerous chronic diseases such as interstitial cystitis, cardiovascular disease, type 2 diabetes, hypertension, cancer, osteoarthritis, and degenerative diseases, including age-associated neurodegenerative disorders, as reviewed by Mormone et al. (i.e., Parkinson's disease, Alzheimer's disease, and lateral amyotrophic and multiple sclerosis). In this review, the authors wanted to summarize the current knowledge on how SIRTs-dependent modulation of the mitochondrial metabolism could impact neurogenesis and neurodegeneration, focusing mainly on the function of ROS and their role in SIRTs-mediated cell reprogramming and telomere protection.

ROS also affect the production, assembly, and turnover of the extracellular matrix (ECM) during wound healing and matrix remodeling. Pathological changes of ROS levels lead to excess ECM production and increased tissue contraction in fibrotic disorders or desmoplastic tumors. On the other hand, the extracellular matrix can modulate ROS production through stimulation of well-known longevity pathways, such as mTOR, FOXO, Nrf2, and sirtuins, whose dysregulation drive the progression of age-related diseases. Within the ECM, hyaluronic acid (HA) constitutes an extracellular scaffold that coordinates the attachment of other ECM components such as proteoglycans and hyaladherins comprising cell surface receptors. The interactions of HA with cell surface receptors induce numerous intracellular signaling pathways (including Nrf2) that act on cell proliferation, migration, and differentiation, depending on its molecular weight. HA is abundant in the brain; it is synthesized by NSCs and increases in the NSCs niches with aging. NSCs express the transmembrane HA receptor CD44, through which HA regulates NSCs neurogenesis. HA metabolism can be modulated together with collagen metabolism by molecules such as those found in pomegranate, a polyphenol-rich fruit; polyphenols are an important modulator of sirtuins. In the review published in this Research Topic, the authors brought together the recent findings on the numerous properties found in this fruit in the treatment of age-related neurological disorders such as Alzheimer's disease and multiple sclerosis, based on its antioxidant and anti-inflammatory properties (Emami Kazemabad et al.).

Understanding what triggers metabolic reprogramming and how cell metabolism directs NSCs fate decisions, together with the ECM interplay, may provide new insight into the brain's regenerative potential. In this context, we think that a deeper knowledge of the molecular mechanism underlying the ROS-dependent regulation of longevity pathways, as a response to cellular redox homeostasis alterations, would be of great help in order to understand how to modulate endogenous neurogenesis through molecules such as polyphenols, photobiomodulation, or through the paracrine effects of stem cells- and extracellular vesicles-based therapy (Figure 1). In fact, their effects may enhance endogenous neuroplasticity and protect existing healthy cells from further damage, such as that of inflammation, while differentiated cells may repair the injured tissue by replacing the damaged or lost cells. This knowledge could help the study of neurodegenerative diseases in the search for sustainable solutions, where regenerative medicine, from an integrated and multidisciplinary perspective, is a candidate to play a crucial role.

**Figure 1:**
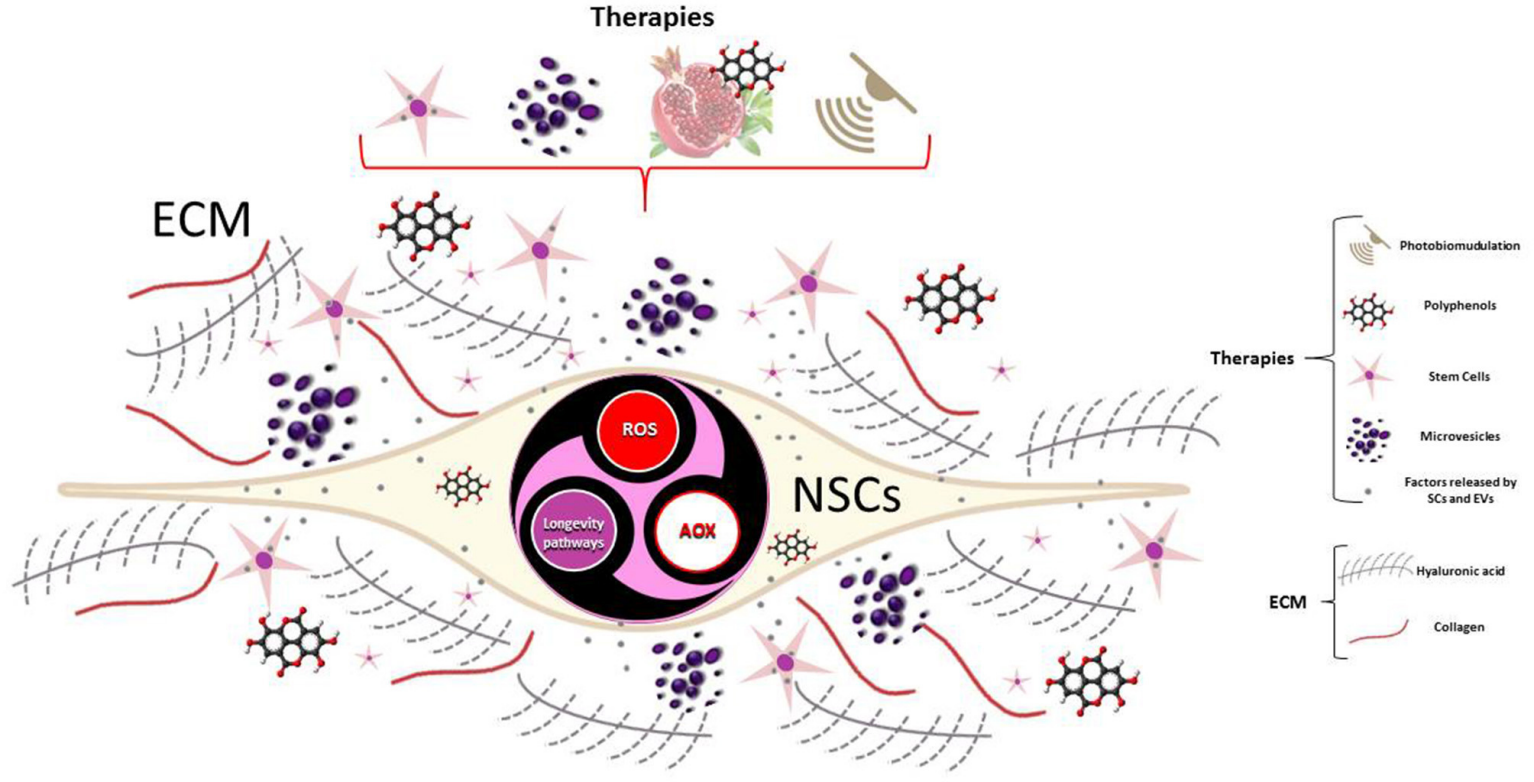
ROS-dependent regulation of longevity pathways can be modulated by stem cell-based and EVs-based therapy, molecules as polyphenols or photobiomodulation, to regulate neurogenesis of NSCs.

## Author contributions

Conceptualization and writing: EM. Review and editing: EM and ELI. All authors contributed to the article and approved the submitted version.

